# Opioid Properties of Nitrous Oxide and Ketamine Contribute to Their Antidepressant Actions

**DOI:** 10.1093/ijnp/pyab045

**Published:** 2021-07-01

**Authors:** Mark A Gillman

**Affiliations:** SA Brain Research Institute, Johannesburg, South Africa

**Significance Statement**

Lately there has been an uptick in interest in a field that has lain dormant for 30 years, that is, examining the psychotropic actions of non-anesthetic concentrations of nitrous oxide (N_2_O). In earlier work, nitrous oxide was shown to be an opioid agonist, but in common with the classical opioid morphine, to interact with other neurotransmitter systems as well. Although there is little doubt that N_2_O at subanaesthetic doses interacts with the opioid system, the recent entrants into the field do not seem to accept the possibility of opioid system involvement in the psychotropic actions of N_2_O. Instead, they suggest that the psychotropic actions are mediated, like ketamine, mainly by the NMDA system alone. There is now definitive evidence from research in animals and man that the antidepressant properties of ketamine are mediated, at least partially, by the opioid system. It is therefore important to emphasise the possibility that like ketamine, the antidepressant actions of subanaesthetic N_2_O is mediated by other neurotransmitters apart from N-methyl-D-aspartate system. My letter presents some of the extant evidence that the gas exerts some of its actions via interactions with the opioid system, as does ketamine.

I welcome the paper by Kamboj et al. (2021), which continues the recent upsurge of research into the psychotropic properties of non-anaesthetic nitrous oxide (N_2_O). However, I would like to comment on a number of matters raised in their paper (Kamboj et al., 2021).

First, I disagree with the notion, held by these workers and most current investigators in the field, that the psychotropic properties of ketamine and subanaesthetic N_2_O are mainly produced by N-Methyl-D-Aspartate receptor (NMDAR) antagonism (for references, see Kamboj et al., 2021). While there is evidence that both N_2_O and ketamine interact with the NMDAR, there is compelling evidence that both substances also interact with opioid receptors (Smith et al., 1987; Sarton et al., 2001; Matussek, 2003; Emmanouil and Quock, 2007; for additional references, see Gillman, 2019a, 2019b) to exert their psychotropic actions. Among the most convincing of these is that blockade of opioid receptors interferes with the antidepressant action of ketamine in animals (Klein et al., 2020) and humans (Williams et al., 2018).

Further, the NMDAR receptor activity of N_2_O occurs not at subanesthetic but predominantly at anesthetic concentrations (Jevtović-Todorović et al., 1998), making it unlikely that the psychotropic actions observed at subanesthetic concentrations are mediated by NMDAR.

Another factor is that memantine, a NMDAR blocker, has little antidepressant or opioid activity (Gillman, 2019b). It is therefore doubtful that the antidepressant actions of ketamine and N_2_O are mainly due to NMADR blockade (Gillman, 2019b), because if this were correct, then memantine, which lacks opioid activity, would also probably have antidepressant properties.

It is worth noting here that in the 19th century, opium was used to ameliorate depression, and more recently, buprenorphine has been used for the same purpose (for references, see Matussek, 2003). Further, upregulation of opioid receptors has been observed in the post-mortem brains of individuals who have committed suicide (Matussek, 2003). Both these facts give more support to opioid involvement in the antidepressant activity of subanaesthetic N_2_O and ketamine (Matussek, 2003; Gillman, 2019a, 2019b).

Moreover, in humans, high impulsivity has been positively correlated with high opioid receptor concentrations in the brain, particularly in regions important for motivational behavior and substance abuse (Love et al., 2009). Significantly, Kamboj et al. (2021) observed a positive correlation between liking for N_2_O and impulsivity. Since subanaesthetic N_2_O and ketamine have opioid properties (Smith et al., 1987; Sarton et al., 2001; Matussek, 2003; Emmanouil and Quock, 2007; for additional references, see Gillman, 2019a, 2019b), these observations (Kamboj et al., 2021) are more likely to have been due to opioid activity rather than NMDAR blockade.

Second, the assumption that the antidepressant actions of subanaesthetic N_2_O was discovered in 2015 is simply wrong. Gillman and Lichtigfeld observed the antidepressant action of subanaesthetic N_2_O in 1982 in alcoholic and later in non-alcoholic individuals in 1985 (see Matussek, 2003; Gillman, 2019a for references).

Third, these researchers (Kamboj et al., 2021) state N_2_O has “also been tested as a treatment for acute alcohol withdrawal.” This may give the wrong impression. Not only has it been tested (Gillman et al., 2007), but it has been widely and successfully used to treat alcoholic withdrawal states in South Africa and Finland, “and in South Africa. . .it is recognised” by the health authorities as a “treatment for substance abuse withdrawal states” (Gillman et al., 2007).

Finally, these workers noted possible blinding problems with their study (Kamboj et al., 2021). To overcome the difficulty of blinding, when using a rapidly acting agent such as N_2_O, Lichtigfeld and I developed a double-blind method used in 2002 and 2004 (see references in Gillman et al., 2007). The latter method enabled us to test double-blind, active diazepine tablets plus placebo gas against placebo tablets and active gas. Consequently, we were able to show double blind that N_2_O is better than diazepam for alcohol withdrawal states. Perhaps these investigators (Kamboj et al., 2021) and others could use a similar method or modification of this method so they can ensure blinding in future investigations of the psychotropic properties of subanaesthetic N_2_O.

## Statement of Interest

I have been researching the psychotropic actions of non-anaesthetic nitrous oxide for over 40 years. Since 2003, I have been the medical adviser to Sedatek, a company that supplies equipment for administering nitrous oxide/oxygen for conscious sedation in South Africa. I own no shares in Sedatek.
